# Perturbation of the Monocyte Compartment in Human Obesity

**DOI:** 10.3389/fimmu.2019.01874

**Published:** 2019-08-08

**Authors:** Kathleen Friedrich, Miriam Sommer, Sarah Strobel, Stephan Thrum, Matthias Blüher, Ulf Wagner, Manuela Rossol

**Affiliations:** ^1^Rheumatology Unit, Department of Medicine, University of Leipzig, Leipzig, Germany; ^2^Division of Endocrinology, Department of Medicine, University of Leipzig, Leipzig, Germany

**Keywords:** obesity, monocytes, myeloid suppressor cells (MDSC), subpopulation, CD16, CD56, macrophages

## Abstract

Circulating monocytes can be divided into classical (CM), intermediate (IM), and non-classical monocytes (NCM), and the classical monocytes also contain CD56+ monocytes and monocytic myeloid-derived suppressor cells (M-MDSC). The aim of the study was to evaluate the occurrence of the monocyte subpopulations in human obesity. Twenty-seven normal, 23 overweight, and 60 obese individuals (including 17 obese individuals with normal glucose tolerance and 27 with type 2 diabetes) were included into this study. Peripheral blood mononuclear cells were isolated from human blood, and surface markers to identify monocyte subpopulations were analyzed by flow cytometry. Obese individuals had higher numbers of total monocytes, CM, IM, CD56+ monocytes, and M-MDSCs. The number of CM, IM, CD56+ monocytes, and M-MDSCs, correlated positively with body mass index, body fat, waist circumference, triglycerides, C-reactive protein, and HbA1c, and negatively with high-density lipoprotein cholesterol. Individuals with obesity and type 2 diabetes had higher numbers of IM, NCM, and M-MDSCs, whereas those with obesity and impaired glucose tolerance had higher numbers of CD56+ monocytes. In summary, the comprehensive analysis of blood monocytes in human obesity revealed a shift of the monocyte compartment toward pro-inflammatory monocytes which might contribute to the development of low-grade inflammation in obesity, and immune-suppressive monocytes which might contribute to the development of cancer in obesity.

## Introduction

Obesity is one of the most prevalent diseases worldwide and increases the risk of developing metabolic and cardiovascular diseases such as type 2 diabetes mellitus (T2D), fatty liver disease, hypertension, coronary heart disease, and some types of cancer (1). Adipose tissue macrophages (ATMs) accumulate in adipose tissue from individuals with obesity and are the major source of inflammatory mediators, leading to obesity-associated chronic tissue inflammation, insulin resistance, and T2D (2, 3). The migration of peripheral blood monocytes contributes to macrophage accumulation in adipose tissue (2, 4–6) but also to the proliferation of resident macrophages (7–9). Wouters et al. reported an association of circulating classical monocytes with the macrophage content of human visceral adipose tissue (6). The chemoattractant monocyte chemoattractant protein-1 (MCP-1) and its receptor C-C chemokine receptor type 2 (CCR2) play an important role in the recruitment of blood monocytes into adipose tissue, where the monocytes mature into macrophages and contribute to the inflammation of the adipose tissue (4, 10, 11). In addition, ATMs promote myelopoiesis and monocytosis via the production of IL-1β (5) which leads to increased leukocyte and monocyte numbers in the peripheral blood of obese individuals (12–14).

Peripheral blood monocytes are not a homogenous cell population, three major subpopulations have been identified: classical monocytes (CD14^bright^/CD16-), intermediate monocytes (CD14^bright^/CD16+) and non-classical monocytes (CD14^dim^/CD16+) (15). Classical monocytes express CCR2, egress from the bone marrow, circulate for 1 day, and then leave the circulation (16). Only a minor proportion of classical monocytes differentiates into intermediate monocytes (4 days in circulation), and most of the intermediate monocytes finally mature into non-classical monocytes (7 days in circulation) (16). CD14^bright^/CD56+ monocytes are a subpopulation within the classical monocytes but this subpopulation is less well-characterized (17, 18). CD56+ monocytes are expanded in autoimmune diseases like rheumatoid arthritis and Crohn's disease, they produce more reactive oxygen intermediates and pro-inflammatory cytokines, they are part of the classical monocyte subpopulation, and more efficient antigen-presenting cells (17–19).

There are only a few studies on the three major monocyte subpopulations in human obesity and the results vary. Rogacev et al. reported about an association of body mass index (BMI) with non-classical monocytes (20), Mattos et al. showed an increased frequency of non-classical monocytes in obese children (21), and Poitou et al. also showed an expansion of the non-classical monocytes in obese individuals but also an increase in intermediate monocytes (22). In contrast, Schipper et al. showed a correlation of BMI with classical and intermediate monocytes in obese children (23). No data on the CD56+ monocyte subpopulation in obesity are available to date.

Monocytic myeloid-derived suppressor cells (M-MDSC) are monocytes with immune-suppressive function, they are expanded in cancer, various autoimmune diseases and in chronic inflammation, and they are characterized by the low expression of HLA-DR (24). The role of M-MDSCs in tumor progression (25, 26) might be of importance in obesity as obesity is associated with an increased cancer risk (27–29). However, there are only a few studies on M-MDSCs in obesity (30–32).

The aim of this study was to evaluate the occurrence of the three major monocyte subpopulations classical monocytes, intermediate monocytes and non-classical monocytes, the CD56+ monocyte subpopulation, and monocytic myeloid-derived suppressor cells in human obesity.

## Materials and Methods

### Study Design and Individuals

Individuals with obesity (BMI ≥ 30 kg/m^2^) were recruited from the Integrated Research and Treatment Center Adiposity Diseases of the Medical Faculty of the University Leipzig and the University Hospital Leipzig. A total of 110 participants, 27 normal, 23 overweight, and 60 obese individuals were included into the study. The classification of normal, overweight, and obese was done according to the definition of the World Health Organization (WHO) based on the body mass index (BMI; body weight in kilograms, divided by height in meters squared; normal BMI 18.5–24.9; overweight BMI 25.0–29.9; obese BMI above 30). The study participants were older than 18 years of age.

The mean BMI of normal individuals was 21.7 kg/m^2^ (25 women and two men, mean age 43.4 years), of the overweight individuals 27.2 kg/m^2^ (21 women and two men, mean age 46.0 years), and of the obese individuals 46.9 kg/m^2^ (50 women and 10 men, mean age 46.4 years).

Determination of clinical laboratory variables were performed at the Institute of Laboratory Medicine, Clinical Chemistry and Molecular Diagnostics, University of Leipzig. Measurement of C-reactive protein (CRP), HbA1c (whole blood), serum total cholesterol, HDL-cholesterol, as well as triglycerides, was performed according to manufacturer's protocol on an automated laboratory analyzer Cobas 8000 (Roche Diagnostics, Mannheim, Germany). Absolute leukocyte and absolute monocyte numbers were determined according to manufacturer's protocol on an automated laboratory analyzer XN-9000 (Sysmex, Norderstedt, Germany).

A 75 g, 2 h, oral glucose tolerance test (OGTT) was performed according to the WHO criteria.

Obese individuals were categorized according to the glycemic status into groups with impaired glucose tolerance (IGT; 36 patients, OGTT > 140 mg/dl, including patients with T2D) and with normal glucose tolerance (17 patients, OGTT < 140 mg/dl). Patients with T2D (27 patients), were classified according to the criteria of the American Diabetes Association (HbA1c levels > 48 mmol/mol and/or OGTT > 200 mg/dl). Thirty-three patients without T2D were included into the study.

Blood samples for peripheral blood mononuclear cell (PBMC) isolation and clinical characteristics (Table 1) were taken in the fasted state. Weight and height of the participants were measured and the waist circumference was taken at the smallest circumference between rib cage and the iliac crest with a standing subject.

**Table 1:** Clinical characteristics of the study participants.

	**Normal**	**Overweight**	**Obese**	***P*-value**
No. (Sex, F/M)	27 (25/2)	23 (21/2)	60 (50/10)	ND
Age, y	43.4 ± 3.0	46.0 ± 2.6	46.4 ± 1.9	NS
Body weight, kg	62.2 ± 1.5	77.4 ± 1.8	134.7 ± 4.4	<0.0001
BMI, kg/m^2^	21.7 ± 0.4	27.2 ± 0.3	46.9 ± 1.1	<0.0001
Fat mass, %	24.1 ± 1.2	32.2 ± 1.3	52.8 ± 1.1^+^	<0.0001
Waist circumference, cm	74.0 ± 1.7	88.9 ± 1.9	129.8 ± 2.4^*^	<0.0001
HbA1c-IFCC, mmol/mol	32.7 ± 0.5	33.9 ± 0.8	43.5 ± 1.6	<0.0001
T2D, n/y	27/0	23/0	33/27	ND
IGT (n/y)	ND	ND	17/36^#^	ND
Total cholesterol, mmol/L	5.2 ± 0.2	5.4 ± 0.2	5.2 ± 0.1	NS
Triglycerides, mmol/L	1.0 ± 0.1	1.3 ± 0.1	2.3 ± 0.3	<0.0001
HDL-cholesterol, mmol/L	1.9 ± 0.1	1.6 ± 0.1	1.3 ± 0	<0.0001
CRP, mg/L	1.2 ± 0.3	1.6 ± 0.3	10.4 ± 0.9	<0.0001
Leukocytes, exp9/L	5.2 ± 0.2	6.1 ± 0.3	8.2 ± 0.3	<0.0001
Monocytes, exp9/L	0.41 ± 0.02	0.48 ± 0.03	0.58 ± 0.02	<0.0001

All values are expressed as mean ± SEM. Statistics were performed as described in Materials and Methods (one-way ANOVA or Kruskal-Wallis test). ^+^Body fat values of two individuals were not available. ^*^Waist circumference values of nine obese individuals were not available. ^#^Data were not available for seven obese individuals. ND not determined, NS not significant, IGT impaired glucose tolerance.

Single frequency bioelectrical impedance analysis (SF-BIA) was used to assess the body composition of all participants. The whole body impedance measurement technique hand to foot was conducted and analyzed with BodyComposition V 9.0 Professional (Software BodyComposition, MEDI Cal HealthCare).

The experimental design of the clinical study has been approved by the ethics committee of the University of Leipzig. Informed and written consent was obtained from all individuals before the enrollment to the study.

### Materials

Flow cytometry antibodies fluorescein isothiocyanate (FITC)-conjugated anti-CD14 (clone TÜK4), phycoerythrin (PE)-conjugated anti-CD16 (clone REA423), allophycocyanin (APC)-conjugated anti-CD56 (clone REA196) and APC-conjugated anti-HLA-DR (clone AC122) and appropriate isotype controls were obtained from Miltenyi Biotec.

### PBMC Isolation

Human peripheral blood mononuclear cells (PBMCs) were isolated by Ficoll-Paque (GE Healthcare Life Sciences) density gradient centrifugation and washed in PBS containing EDTA.

### Flow Cytometry

PBMCs (1 × 10^6^/100 μl) were stained with CD14-FITC, CD16-PE, CD56-APC antibodies, and anti-HLA-DR-APC antibody and isotype controls for 20 min at 4°C. Cells were washed twice with PBS supplemented with 2% FCS and 0.1% sodium azide and fixed with 3% formaldehyde. Samples were measured using the BD LSR II and analyzed using FlowJo Version 8.7 (Tree Star) software. Gating strategies to identify the monocyte subpopulations are shown in Supplementary Figures 1, 2. Monocyte subpopulation numbers were calculated using following equation: (absolute monocyte number ^*^ percentage of monocyte subpopulation)/100.

### Statistical Analysis

For statistical analysis GraphPad PRISM Version 5 (GraphPad Software Inc., San Diego, CA, USA) was used. First, a normality test was performed for all comparisons and statistical significances were evaluated by Student's *t*-test or the Mann-Whitney *U* rank sum test. One-way ANOVA or Kruskal-Wallis test was conducted for the comparison of more than two groups.

To assess the correlation between two variables Person‘s correlation coefficient for normally distributed data or Spearman‘s rank correlation coefficient for not normally distributed data was used. To assess whether the association of CD56+ monocytes and of M-MDSC with clinical and laboratory parameters is independent of the association with classical monocytes, we adjusted for classical monocytes.

## Results

### The CD14/CD16 Monocyte Subpopulations in Obesity

The clinical characteristics of normal, overweight, and obese individuals are presented in Table 1. Sixty obese individuals, 23 overweight individuals, and 27 normal individuals were recruited. In addition to higher BMI, obese individuals showed higher fat mass, waist circumference; deterioration of metabolic variables, such as lipid variables and HbA1c-IFCC; and an increased number of leukocytes and monocytes. The CRP levels were significantly higher in obese individuals compared to the other BMI categories.

**Figure 1:**
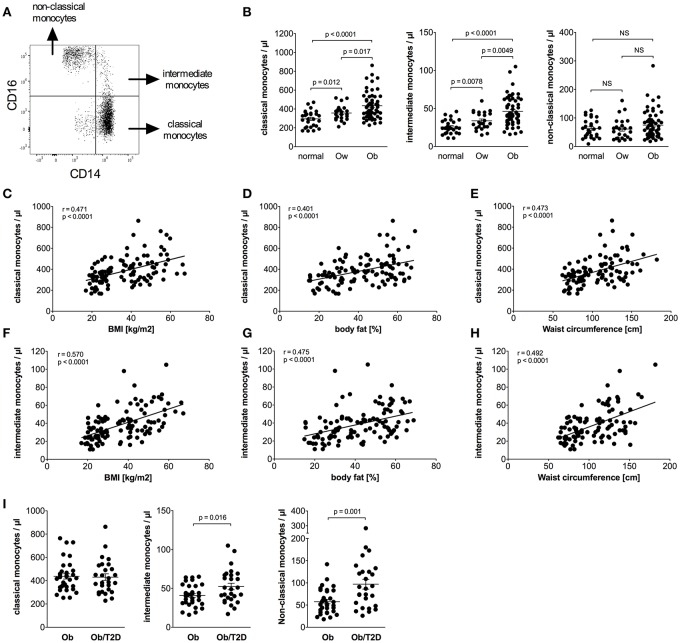
Quantification of classical, intermediate, and non-classical monocytes of normal, overweight, and obese individuals. **(A)** Representative dot plot of CD14 and CD16 expression on monocytes. **(B)** Absolute numbers of classical, intermediate, and non-classical monocytes of normal (*n* = 27), overweight (ow, *n* = 23), and obese (ob, *n* = 60) individuals. Scatter plots show mean ± SEM. **(C–E)** Correlation of classical monocyte numbers with BMI (**C**, *n* = 110), body fat (**D**, *n* = 108), and waist circumference (**E**, *n* = 101). **(F–H)** Correlation of intermediate monocyte numbers with BMI (**F**, *n* = 110), body fat (**G**, *n* = 108), and waist circumference (**H**, *n* = 101). **(I)** Absolute numbers of classical, intermediate, and non-classical monocytes of obese individuals with (Ob/T2D, *n* = 27) and without (Ob, *n* = 33) type 2 diabetes. Scatter plots show mean ± SEM.

Calculation of absolute numbers of monocyte subsets revealed that obese individuals have expanded classical monocytes and intermediate monocytes in comparison to normal and overweight individuals (Figure 1B). In contrast, non-classical monocyte numbers were indistinguishable between obese, overweight, and normal individuals (Figure 1B). Obese individuals had a higher percentage of intermediate monocytes in total monocytes than normal individuals (8.1% ± 0.4 vs. 6.2% ± 0.3, *p* = 0.001), and equal percentages of classical monocytes (75.1% ± 1.1 vs. 71.6% ± 1.9, NS), and non-classical monocytes (13.0% ± 0.9 vs. 15.8% ± 1.5, NS).

Detailed analysis of clinical variables in the entire cohort (Table 2) revealed associations between classical monocyte numbers and variables of obesity. There were associations between the absolute number of classical monocytes and BMI, waist circumference, and fat mass (Figures 1C–E). The number of intermediate monocytes also strongly correlated with BMI (Figure 1F), fat mass (Figure 1G), and waist circumference (Figure 1H). Classical and intermediate monocyte numbers were associated with triglyceride and HDL-c values, more strongly with intermediate monocyte numbers (Table 2). The inflammation marker CRP was strongly associated with both classical and intermediate monocyte numbers. Stratification of obese individuals for CRP levels showed that obese individuals with clinically raised CRP levels (>5 mg/L) and obese individuals with <5 mg/L CRP had equal numbers of classical (442/μl ± 23 vs. 414/μl ± 24, NS), intermediate (47/μl ± 3 vs. 43/μl ± 5, NS), and non-classical monocytes (79/μl ± 8 vs. 65/μl ± 10, NS). Obese individuals with CRP levels >5 mg/L and obese individuals with CRP < 5 mg/L had significantly more classical and intermediate monocytes than normal individuals (data not shown).

**Table 2:** Correlations between monocyte subsets and variables of obesity, glucose, lipid metabolism, and inflammation in the entire cohort.

	**Classical monocytes**		**Intermediate monocytes**	
	***r***	***P*-value**	***r***	***P*-value**
BMI	0.473	<0.0001	0.570	<0.0001
Fat mass, %	0.401	<0.0001	0.475	<0.0001
Waist	0.473	<0.0001	0.492	<0.0001
HbA1c-IFCC	0.261	0.0059	0.473	<0.0001
Total Cholesterol	−0.09	NS	0.01	NS
Triglycerides	0.253	0.0078	0.489	<0.0001
HDL-c	−0.234	0.0140	−0.294	0.0018
CRP	0.406	<0.0001	0.453	<0.0001

*n = 110. Data show Pearson or Spearman correlation coefficient. Residual plots of the data are shown in Supplementary Figures 3, 4. Statistics were performed as described in Materials and Methods. NS, not significant*.

Glycosylated hemoglobin (HbA1c) correlated with classical monocyte numbers and more strongly with intermediate monocyte numbers (Table 2). Stratification of obese individuals into those with or without T2D did not reveal a significant difference in classical monocyte numbers between both groups. However, increased intermediate monocyte and non-classical monocyte numbers were observed in obese individuals with T2D (Figure 1I). Obese individuals with and without impaired glucose tolerance have equal classical, intermediate and non-classical monocyte numbers (data not shown).

### The CD14/CD56 Monocyte Subpopulation in Obesity

**Figure 2:**
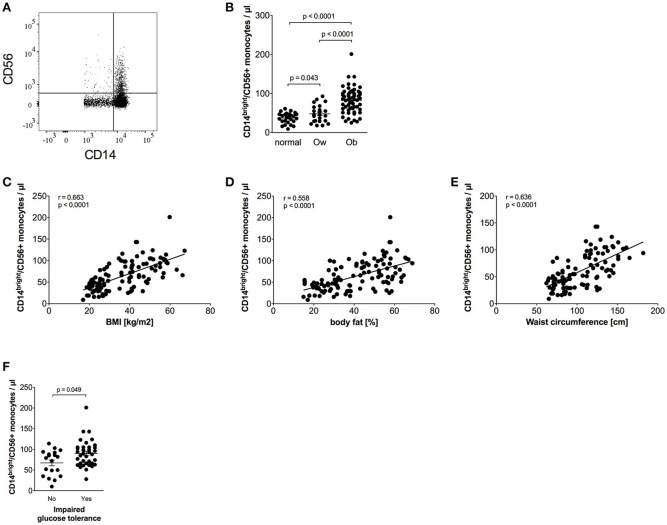
Quantification of CD56+ monocytes of normal, overweight, and obese individuals. **(A)** Representative dot plot of CD14 and CD56 expression on monocytes. **(B)** Absolute numbers of CD56+ monocytes of normal (*n* = 27), overweight (ow, *n* = 23), and obese (ob, *n* = 60) individuals. Scatter plots show mean ± SEM. **(C–E)** Correlation of CD56+ monocyte numbers with BMI (**C**, *n* = 110), body fat (**D**, *n* = 108), and waist circumference (**E**, *n* = 101). **(F)** Absolute numbers of CD56+ monocytes of obese individuals with (*n* = 36) and without (*n* = 19) impaired glucose tolerance. Scatter plots show mean ± SEM.

CD56+ monocytes were identified using CD14/CD56 staining, as shown in Figure 2A. Obese individuals had a higher percentage of CD14^bright^/CD56+ monocytes in total monocytes than normal individuals (14.4% ± 0.6 vs. 9.2% ± 0.7, *p* < 0.0001) and overweight individuals (14.4% ± 0.6 vs. 10.3% ± 1.1, *p* = 0.0005). The percentage of CD14^bright^/CD56+ monocytes among classical monocytes of obese individuals was also increased compared to normal individuals (19.5% ± 0.9 vs. 12.9% ± 0.9, *p* < 0.0001) and overweight individuals (19.5% ± 0.9 vs. 13.7% ± 1.3, *p* = 0.0005).

The calculated absolute number of CD14^bright^/CD56+ monocytes was also increased in obese individuals compared to both normal and overweight individuals (Figure 2B). We have previously shown that the majority of CD56+ monocytes co-expresses CD14^bright^ and is CD16 negative, and is therefore a subpopulation of classical monocytes (17). This could be confirmed for normal, overweight, and obese individuals in this cohort (data not shown). The frequency of CD56+ monocytes expands with age in healthy controls, as we have shown in a previous study (17). However, analysis of the combined cohort of normal, overweight, and obese individuals did not show an association of age and the absolute number of CD56+ monocytes or the frequency of CD56+ monocytes (data not shown).

Detailed analysis of clinical variables in the whole cohort (Table 3) revealed associations of CD14^bright^/CD56+ monocyte numbers and CD56+ numbers adjusted for classical monocytes with variables of corpulence. There were strong associations between the absolute number of CD14^bright^/CD56+ monocytes and BMI (Figure 2C), fat mass (Figure 2D), and waist circumference (Figure 2E). In addition, there were correlations with lipid variables (triglycerides, HDL-c) and a strong positive correlation with CRP. There was no difference in the numbers of CD56+ monocytes between individuals with obesity and CRP levels >5 mg/L or <5 mg/L (87/μl ± 5 vs. 72 ± 5, NS). HbA1c strongly correlated with CD56+ monocyte numbers. Stratification of obese individuals in individuals with and without diabetes did not reveal significant differences in CD56+ monocyte numbers (data not shown). However, there were higher CD56+ monocyte numbers in obese individuals with impaired glucose tolerance in comparison to obese individuals with normal glucose tolerance (Figure 2F).

**Table 3:** Correlations between CD56+ monocytes and variables of obesity, glucose, lipid metabolism, and inflammation in the entire cohort.

	**CD56+** **monocytes**		**CD56+** **monocytes adjusted for classical monocytes**	
	***r***	***P*-value**	***r***	***P*-value**
BMI	0.663	<0.0001	0.425	<0.0001
Fat mass, %	0.558	<0.0001	0.332	<0.0001
Waist	0.636	<0.0001	0.389	<0.0001
HbA1c-IFCC	0.603	<0.0001	0.556	<0.0001
Total Cholesterol	0.004	NS	0.106	NS
Triglycerides	0.414	<0.0001	0.315	0.001
HDL-c	−0.366	<0.0001	−0.274	0.004
CRP	0.585	<0.0001	0.376	<0.0001

*n = 110. Data show Pearson or Spearman correlation coefficient. Residual plots of the data are shown in Supplementary Figure 5. Statistics were performed as described in Materials and Methods. NS, not significant*.

### The M-MDSC Monocyte Subpopulation in Obesity

**Figure 3:**
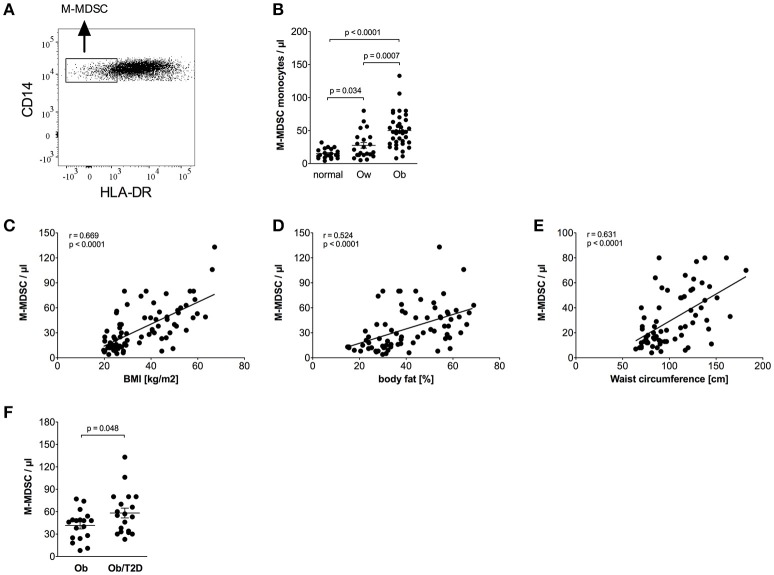
Quantification of M-MDSCs of normal, overweight, and obese individuals. **(A)** Representative dot plot of CD14 and HLA-DR expression on monocytes. **(B)** Absolute numbers of M-MDSCs of normal (*n* = 21), overweight (ow, *n* = 22), and obese (ob, *n* = 37) individuals. Scatter plots show mean ± SEM. **(C–E)** Correlation of M-MDSC numbers with BMI (**C**, *n* = 80), body fat (**D**, *n* = 75), and waist circumference (**E**, *n* = 67). **(F)** Absolute numbers of M-MDSCs of obese individuals with (*n* = 19) and without (*n* = 18) impaired glucose tolerance. Scatter plots show mean ± SEM.

Monocytic myeloid-derived suppressor cells (M-MDSCs) were identified in classical monocytes using CD14/HLA-DR staining, as shown in Figure 3A. M-MDSCs are defined by high CD14 and low HLA-DR expression (24). Intermediate and non-classical monocytes were excluded because they express high amounts of HLA-DR (33, 34). Obese individuals had a higher percentage of M-MDSC monocytes in the classical monocyte subset than normal individuals (12.0% ± 1.0 vs. 5.4% ± 0.5, *p* < 0.0001) and overweight individuals (12.0% ± 1.0 vs. 7.4% ± 1.1, *p* = 0.0015). The calculated absolute number of M-MDSC monocytes was also increased in obese individuals compared to normal controls and overweight individuals (Figure 3B).

Detailed analysis of clinical variables in the whole cohort (Table 4) revealed associations of M-MDSC numbers and M-MDSC numbers adjusted for classical monocytes with variables of obesity. There were strong associations between the absolute number of M-MDSCs and BMI (Figure 3C), fat mass (Figure 3D), and waist circumference (Figure 3E). There was a correlation with triglycerides, HDL-c and a strong positive correlation with CRP. Obese individuals with CRP levels >5 mg/L were not different from those with CRP <5 mg/L with regard to the numbers of M-MDSCs (55/μl ± 6 vs. 41 ± 5, NS).

**Table 4:** Correlations between M-MDSC monocytes and variables of obesity, glucose, lipid metabolism, and inflammation in the entire cohort.

	**M-MDSC**		**M-MDSC adjusted for classical monocytes**	
	***r***	***P* value**	***r***	***P* value**
BMI	0.669	<0.0001	0.393	<0.0001
Fat mass, %	0.524	<0.0001	0.287	0.011
Waist	0.631	<0.0001	0.340	0.004
HbA1c-IFCC	0.470	<0.0001	0.274	0.014
Total Cholesterol	−0.154	NS	0.056	NS
Triglycerides	0.369	0.0008	0.272	0.014
HDL-c	−0.419	0.0001	−0.349	0.002
CRP	0.569	<0.0001	0.424	<0.0001

*n = 80. Data show Pearson or Spearman correlation coefficient. Residual plots of the data are shown in Supplementary Figure 6. Statistics were performed as described in Materials and Methods. NS, not significant*.

HbA1c correlated with M-MDSC numbers (Table 4). Stratification of obese individuals in individuals with and without impaired glucose tolerance did not reveal differences in M-MDSC numbers (data not shown). However, there were increased M-MDSC numbers in obese individuals with T2D in comparison to obese individuals without T2D (Figure 3F).

## Discussion

**Figure 4:**
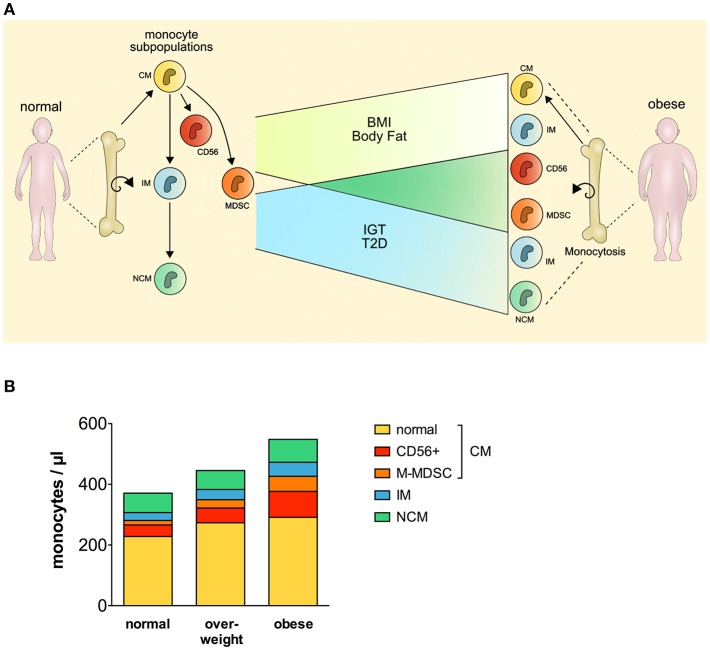
Perturbation of the monocyte compartment in human obesity. CM, classical monocytes; IM, intermediate monocytes; NCM, non-classical monocytes; MDSC, myeloid derived suppressor cells; BMI, body mass index; IGT, impaired glucose tolerance; T2D, type 2 diabetes.

Our findings suggest that classical monocytes, intermediate monocytes, CD56+ monocytes, and M-MDSCs but not non-classical monocytes, are expanded and contribute to monocytosis in human obesity (Figure 4A). Newly released monocytes from the bone marrow are classical monocytes, they represent ~ 80% of the monocytes in the peripheral blood (16). Most cells of this monocyte subset leave the circulation after 1 day, and only a minor proportion of classical monocytes matures into intermediate monocytes and subsequently into non-classical monocytes (16). This goes in line with our finding that classical monocytes are present with increased percentage in the blood of obese individuals, however, intermediate monocytes were also increased in obese individuals. Increased leukocyte and monocyte numbers in the peripheral blood of obese individuals have been reported before (12–14) and were also observed in our study. Nagareddy et al. demonstrated that monocytosis in obesity is due to IL-1β produced by ATMs in adipose tissue, which then stimulates the production of monocytes in the bone marrow (5). As classical monocytes are released from the bone marrow and the other two subpopulations mature subsequently out of classical monocytes, one might expect the increased classical monocyte numbers seen in our study. Interestingly, increased classical monocyte numbers in obese individuals were only observed in one other study (23), the other studies report about increased intermediate or non-classical monocytes (13, 20–22). This might be due to different gating strategies used to discriminate between intermediate and non-classical monocytes. Zawada et al. compared the two gating strategies, the rectangular gating strategy and the trapezoid gating strategy and there was no difference in the results of this study on patients with chronic kidney disease (35). However, the use of the trapezoid gating strategy results in higher numbers of non-classical monocytes and lower numbers of intermediate monocytes compared to the rectangular gating strategy. We used the rectangular gating strategy in this study on obese individuals (Figure 1A) and also in a previous study on rheumatoid arthritis patients (34), mainly because intermediate monocytes are defined as CD14^bright^ and non-classical monocytes as CD14^dim^ and the rectangular gating strategy reflects this better than the trapezoid gating strategy.

CCR2 plays an important role in the recruitment of monocytes into the adipose tissue. Overexpression of monocyte chemoattractant protein-1 (MCP-1), the ligand of CCR2, leads to accumulation of macrophages in the adipose tissue of mice (11), and CCR2 deficiency in mice results in lowered macrophage content of adipose tissue (36). Human classical monocytes are known to have a high expression of CCR2, whereas non-classical monocytes do not express CCR2, and intermediate monocytes express CCR2 but at lower levels than classical monocytes (37). In addition, Devêvre et al. showed that classical and intermediate monocytes of obese individuals expressed more CCR2 than control individuals (13), Krenninger et al. observed increased CCR2 expression on classical monocytes (38), and Pecht et al. demonstrated that non-classical monocytes had the lowest migratory capacity toward adipose tissue conditioned medium compared to classical and intermediate monocytes (39). This might point to a higher capacity of classical and intermediate monocytes to migrate into adipose tissue.

In addition to classical and intermediate monocytes, CD56+ monocytes are also expanded in obese patients. There are only a few studies of this subpopulation. CD56+ monocytes are pro-inflammatory cells, part of the classical monocyte subpopulation, more efficient antigen-presenting cells, and expanded in rheumatoid arthritis and Crohn's disease (17–19). In our study, CD56+ monocytes were strongly associated with higher BMI, body fat, waist circumference, triglycerides, HbA1c, inflammation, and lower HDL-c. CD56+ monocytes were also expanded in overweight individuals in comparison to normal controls, and obese patients with impaired glucose tolerance showed the strongest expansion of CD56+ monocytes. We can only speculate on the function of this subpopulation in obesity, but as CD56+ monocytes are able to produce more pro-inflammatory cytokines and express, as part of the classical monocyte subpopulation, CCR2, they might be able to migrate toward the adipose tissue and contribute to the low-grade inflammation.

The presence of MDSCs in obese patients was hypothesized because the low grade inflammation in obesity, and the obesity related presence of fatty acids supports the induction and accumulation of MDSCs (30, 40–42). There is, to our knowledge, only one study demonstrating the presence of M-MDSCs in human obesity. Bao et al. showed that M-MDSCs are expanded in obese/overweight Chinese men, however, the study cohort was very small, eight normal controls, and eight obese/overweight patients (31). M-MDSCs are also expanded in obese mice (30, 43). Our study demonstrates a strong expansion of M-MDSCs with increasing BMI, body fat, waist circumference, triglycerides, inflammation, and decreasing HDL-c. M-MDSC numbers also expanded with increasing concentrations of glycosylated hemoglobin, and the strongest expansion of MDSCs was seen in obese patients with T2D.

Human M-MDSCs in peripheral blood are currently defined as CD14+/HLA-DR^low/−^, however, there is no specific marker to identify M-MDCSs yet (24). The phenotypic evaluation by flow cytometry is sufficient to analyze the proportion of M-MDSCs in the peripheral blood of controls and patients, but functional studies should succeed to evaluate the immune-suppressive function of the cells (24).

M-MDSCs influence a plethora of innate and adaptive immune responses, most of all studied in relation to cancer (25, 26). Obesity is associated with an increased risk to develop various cancers (27–29), and as M-MDSCs facilitate tumor growth by various mechanisms, the increased M-MDSC numbers in obese patients might contribute to the development of cancer in those patients. This was demonstrated in mice as MDSCs facilitated tumor growth, however, MDSCs also had beneficial effects and protected mice against metabolic dysfunction and inflammation (30).

CD56+ monocytes and M-MDSCs are mainly part of the classical monocytes, and this raised the question whether classical monocytes without those two subpopulations are still expanded in obese individuals. As seen in Figure 4B, CD56+ monocytes and M-MDSCs contribute most to the expansion of classical monocytes, however, classical monocyte numbers without the CD56+ subpopulation and M-MDSCs are also increased in obese individuals compared to normal individuals.

In summary, the comprehensive analysis of blood monocytes in human obesity revealed a perturbation of the monocyte compartment with increased numbers of total monocytes, classical monocytes, intermediate monocytes, CD56+ monocytes, and M-MDSCs. Increased monocyte subpopulation numbers were associated with increasing BMI, body fat, waist circumference, triglycerides, HbA1c, inflammation, and decreasing HDL-c. The deterioration of the monocyte compartment toward pro-inflammatory monocytes and also immune-suppressive monocytes might contribute to the development of low-grade inflammation and cancer in obesity.

## Data Availability

The raw data supporting the conclusions of this manuscript will be made available by the authors, without undue reservation, to any qualified researcher.

## Ethics Statement

The experimental design of the clinical study has been approved by the ethics committee of the University of Leipzig. Informed consent was obtained from all individuals before the enrollment to the study.

## Author Contributions

KF performed the experiments and was involved in data analysis and drafting of the manuscript. SS analyzed data and prepared figures. MS and ST recruited patients and analyzed clinical data. MB recruited patients and was involved in drafting of the manuscript. UW was involved in drafting of the manuscript. MR conceived of the project, involved in data analysis, and drafted the manuscript.

### Conflict of Interest Statement

The authors declare that the research was conducted in the absence of any commercial or financial relationships that could be construed as a potential conflict of interest.

## References

[1] GBD 2015 Obesity Collaborators Afshin A Forouzanfar MH Reitsma MB Sur P Estep K Health effects of overweight and obesity in 195 countries over 25 years. N Engl J Med. (2017) 377:13–27. 10.1056/NEJMoa1614362 28604169 PMC5477817

[2] Weisberg SP McCann D Desai M Rosenbaum M Leibel RL Ferrante AW. Obesity is associated with macrophage accumulation in adipose tissue. J Clin Invest. (2003) 112:1796–808. 10.1172/JCI200319246 14679176 PMC296995

[3] Olefsky JM Glass CK. Macrophages, inflammation, and insulin resistance. Annu Rev Physiol. (2010) 72:219–46. 10.1146/annurev-physiol-021909-135846 20148674

[4] Oh DY Morinaga H Talukdar S Bae EJ Olefsky JM. Increased macrophage migration into adipose tissue in obese mice. Diabetes. (2012) 61:346–54. 10.2337/db11-0860 22190646 PMC3266418

[5] Nagareddy PR Kraakman M Masters SL Stirzaker RA Gorman DJ Grant RW. Adipose tissue macrophages promote myelopoiesis and monocytosis in obesity. Cell Metab. (2014) 19:821–35. 10.1016/j.cmet.2014.03.029 24807222 PMC4048939

[6] Wouters K Gaens K Bijnen M Verboven K Jocken J Wetzels S. Circulating classical monocytes are associated with CD11c^+^ macrophages in human visceral adipose tissue. Sci Rep. (2017) 7:42665. 10.1038/srep42665 28198418 PMC5309742

[7] Amano SU Cohen JL Vangala P Tencerova M Nicoloro SM Yawe JC. Local proliferation of macrophages contributes to obesity-associated adipose tissue inflammation. Cell Metab. (2014) 19:162–71. 10.1016/j.cmet.2013.11.017 24374218 PMC3931314

[8] Zheng C Yang Q Cao J Xie N Liu K Shou P. Local proliferation initiates macrophage accumulation in adipose tissue during obesity. Cell Death Dis. (2016) 7:e2167. 10.1038/cddis.2016.54 27031964 PMC4823955

[9] Haase J Weyer U Immig K Klöting N Blüher M Eilers J. Local proliferation of macrophages in adipose tissue during obesity-induced inflammation. Diabetologia. (2014) 57:562–71. 10.1007/s00125-013-3139-y 24343232

[10] Lumeng CN Deyoung SM Bodzin JL Saltiel AR. Increased inflammatory properties of adipose tissue macrophages recruited during diet-induced obesity. Diabetes. (2007) 56:16–23. 10.2337/db06-1076 17192460

[11] Kamei N Tobe K Suzuki R Ohsugi M Watanabe T Kubota N. Overexpression of monocyte chemoattractant protein-1 in adipose tissues causes macrophage recruitment and insulin resistance. J Biol Chem. (2006) 281:26602–14. 10.1074/jbc.M601284200 16809344

[12] Kullo IJ Hensrud DD Allison TG. Comparison of numbers of circulating blood monocytes in men grouped by body mass index (<25, 25 to <30, > or = 30). Am J Cardiol. (2002) 89:1441–3. 10.1016/S0002-9149(02)02366-4 12062747

[13] Devêvre EF Renovato-Martins M Clément K Sautès-Fridman C Cremer I Poitou C. Profiling of the three circulating monocyte subpopulations in human obesity. J Immunol Baltim Md 1950. (2015) 194:3917–23. 10.4049/jimmunol.1402655 25786686

[14] Vuong J Qiu Y La M Clarke G Swinkels DW Cembrowski G. Reference intervals of complete blood count constituents are highly correlated to waist circumference: should obese patients have their own “normal values?” Am J Hematol. (2014) 89:671–7. 10.1002/ajh.23713 24644218

[15] Ziegler-Heitbrock L Ancuta P Crowe S Dalod M Grau V Hart DN. Nomenclature of monocytes and dendritic cells in blood. Blood. (2010) 116:e74–80. 10.1182/blood-2010-02-258558 20628149

[16] Patel AA Zhang Y Fullerton JN Boelen L Rongvaux A Maini AA. The fate and lifespan of human monocyte subsets in steady state and systemic inflammation. J Exp Med. (2017).20170355. 10.1084/jem.20170355 28606987 PMC5502436

[17] Krasselt M Baerwald C Wagner U Rossol M. CD56+ monocytes have a dysregulated cytokine response to lipopolysaccharide and accumulate in rheumatoid arthritis and immunosenescence. Arthritis Res Ther. (2013) 15:R139. 10.1186/ar4321 24286519 PMC3978677

[18] Sconocchia G Keyvanfar K El Ouriaghli F Grube M Rezvani K Fujiwara H. Phenotype and function of a CD56+ peripheral blood monocyte. Leukemia. (2005) 19:69–76. 10.1038/sj.leu.2403550 15526027

[19] Grip O Bredberg A Lindgren S Henriksson G. Increased subpopulations of CD16^+^ and CD56^+^ blood monocytes in patients with active Crohn's disease. Inflamm Bowel Dis. (2007) 13:566–72. 10.1002/ibd.20025 17260384

[20] Rogacev KS Ulrich C Blömer L Hornof F Oster K Ziegelin M. Monocyte heterogeneity in obesity and subclinical atherosclerosis. Eur Heart J. (2010) 31:369–376. 10.1093/eurheartj/ehp308 19687164

[21] Mattos RT Medeiros NI Menezes CA Fares RC Franco EP Dutra WO. Chronic low-grade inflammation in childhood obesity is associated with decreased IL-10 expression by monocyte subsets. PLoS ONE. (2016) 11:e0168610. 10.1371/journal.pone.0168610 27977792 PMC5158089

[22] Poitou C Dalmas E Renovato M Benhamo V Hajduch F Abdennour M. CD14dimCD16+ and CD14+CD16+ monocytes in obesity and during weight loss: relationships with fat mass and subclinical atherosclerosis. Arterioscler Thromb Vasc Biol. (2011) 31:2322–30. 10.1161/ATVBAHA.111.230979 21799175

[23] Schipper HS Nuboer R Prop S van den Ham HJ de Boer FK Kesmir Ç. Systemic inflammation in childhood obesity: circulating inflammatory mediators and activated CD14++ monocytes. Diabetologia. (2012) 55:2800–10. 10.1007/s00125-012-2641-y 22806355

[24] Bronte V Brandau S Chen SH Colombo MP Frey AB Greten TF. Recommendations for myeloid-derived suppressor cell nomenclature and characterization standards. Nat Commun. (2016) 7:12150. 10.1038/ncomms12150 27381735 PMC4935811

[25] Groth C Hu X Weber R Fleming V Altevogt P Utikal J. Immunosuppression mediated by myeloid-derived suppressor cells (MDSCs) during tumour progression. Br J Cancer. (2018) 120:16–25. 10.1038/s41416-018-0333-1 30413826 PMC6325125

[26] Gabrilovich DI Ostrand-Rosenberg S Bronte V. Coordinated regulation of myeloid cells by tumours. Nat Rev Immunol. (2012) 12:253–68. 10.1038/nri3175 22437938 PMC3587148

[27] Kyrgiou M Kalliala I Markozannes G Gunter MJ Paraskevaidis E Gabra H. Adiposity and cancer at major anatomical sites: umbrella review of the literature. BMJ. (2017) 356:j477. 10.1136/bmj.j477 28246088 PMC5421437

[28] Lauby-Secretan B Scoccianti C Loomis D Grosse Y Bianchini F Straif K. Body fatness and cancer–viewpoint of the IARC working group. N Engl J Med. (2016) 375:794–8. 10.1056/NEJMsr1606602 27557308 PMC6754861

[29] Calle EE Rodriguez C Walker-Thurmond K Thun MJ. Overweight, obesity, and mortality from cancer in a prospectively studied cohort of U.S. adults. N Engl J Med. (2003) 348:1625–38. 10.1056/NEJMoa021423 12711737

[30] Clements VK Long T Long R Figley C Smith DMC Ostrand-Rosenberg S. Frontline science: high fat diet and leptin promote tumor progression by inducing myeloid-derived suppressor cells. J Leukoc Biol. (2018) 103:395–407. 10.1002/JLB.4HI0517-210R 29345342 PMC7414791

[31] Bao Y Mo J Ruan L Li G Increased monocytic CD14+HLADRlow/- myeloid-derived suppressor cells in obesity. Mol Med Rep. (2015) 11:2322–8. 10.3892/mmr.2014.2927 25384365

[32] Hale M Itani F Buchta CM Wald G Bing M Norian LA Obesity triggers enhanced MDSC accumulation in murine renal tumors via elevated local production of CCL2. PLoS ONE. (2015) 10:e0118784 10.1371/journal.pone.0118784 25769110 PMC4358922

[33] Abeles RD McPhail MJ Sowter D Antoniades CG Vergis N Vijay GK. CD14, CD16 and HLA-DR reliably identifies human monocytes and their subsets in the context of pathologically reduced HLA-DR expression by CD14(hi) /CD16(neg) monocytes: expansion of CD14(hi) /CD16(pos) and contraction of CD14(lo) /CD16(pos) monocytes in acute liver failure. Cytom Part J Int Soc Anal Cytol. (2012) 81:823–34. 10.1002/cyto.a.22104 22837127

[34] Rossol M Kraus S Pierer M Baerwald C Wagner U. The CD14(bright) CD16+ monocyte subset is expanded in rheumatoid arthritis and promotes expansion of the Th17 cell population. Arthritis Rheum. (2012) 64:671–7. 10.1002/art.33418 22006178

[35] Zawada AM Fell LH Untersteller K Seiler S Rogacev KS Fliser D. Comparison of two different strategies for human monocyte subsets gating within the large-scale prospective CARE FOR HOMe Study. Cytometry A. (2015) 87:750–8. 10.1002/cyto.a.22703 26062127

[36] Weisberg SP Hunter D Huber R Lemieux J Slaymaker S Vaddi K. CCR2 modulates inflammatory and metabolic effects of high-fat feeding. J Clin Invest. (2006) 116:115–24. 10.1172/JCI24335 16341265 PMC1307559

[37] Hijdra D Vorselaars AD Grutters JC Claessen AM Rijkers GT. Phenotypic characterization of human intermediate monocytes. Front Immunol. (2013) 4:339. 10.3389/fimmu.2013.00339 24155746 PMC3805031

[38] Krinninger P Ensenauer R Ehlers K Rauh K Stoll J Krauss-Etschmann S. Peripheral monocytes of obese women display increased chemokine receptor expression and migration capacity. J Clin Endocrinol Metab. (2014) 99:2500–9. 10.1210/jc.2013-2611 24606068

[39] Pecht T Haim Y Bashan N Shapiro H Harman-Boehm I Kirshtein B. Circulating blood monocyte subclasses and lipid-laden adipose tissue macrophages in human obesity. PLOS ONE. (2016) 11:e0159350. 10.1371/journal.pone.0159350 27442250 PMC4956051

[40] Wu H Weidinger C Schmidt F Keye J Friedrich M Yerinde C Oleate but not stearate induces the regulatory phenotype of myeloid suppressor cells. Sci Rep. (2017) 7:7498 10.1038/s41598-017-07685-9 28790345 PMC5548895

[41] Yan D Yang Q Shi M Zhong L Wu C Meng T. Polyunsaturated fatty acids promote the expansion of myeloid-derived suppressor cells by activating the JAK/STAT3 pathway. Eur J Immunol. (2013) 43:2943–55. 10.1002/eji.201343472 23897117

[42] Ostrand-Rosenberg S. Myeloid derived-suppressor cells: their role in cancer and obesity. Curr Opin Immunol. (2018) 51:68–75. 10.1016/j.coi.2018.03.007 29544121 PMC5943167

[43] Xia S Sha H Yang L Ji Y Ostrand-Rosenberg S Qi L. Gr-1+ CD11b+ myeloid-derived suppressor cells suppress inflammation and promote insulin sensitivity in obesity. J Biol Chem. (2011) 286:23591–9. 10.1074/jbc.M111.237123 21592961 PMC3123122

