# Supplementary material for: Perturbation of the Monocyte Compartment in Human Obesity
Source: Front Immunol. 2019 Aug 8;10:1874. doi: 10.3389/fimmu.2019.01874 (PMC6694869; doi:10.3389/fimmu.2019.01874)
Supplement: Supplementary file 1 [file Data_Sheet_1.PDF]

Supplementary Figure 1:

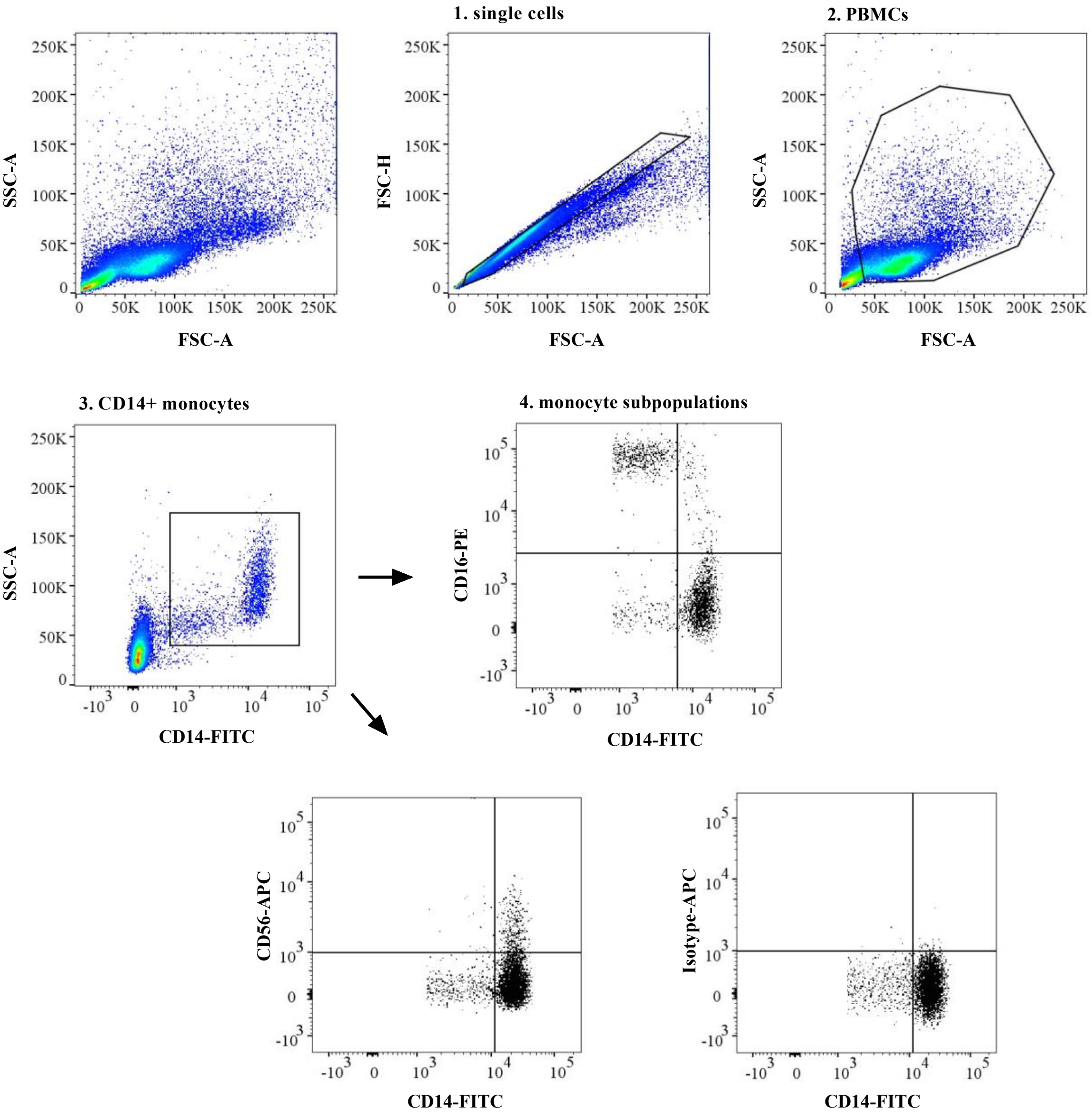

Flow cytometry gating strategy for monocyte subpopulations: 1.single cells were selected and viable PBMCs were plotted according to their physical parameters, forward and sideward light scatter (2). 3. CD14+ monocytes were identified by plotting CD14 against SSC. 4. the subpopulations were determined by the use of different co-stainings of CD14-FITC/ CD16-PE, CD14-FITC/ CD56-APC and CD14-FITC/ Isotype-APC.

Supplementary Figure 2:

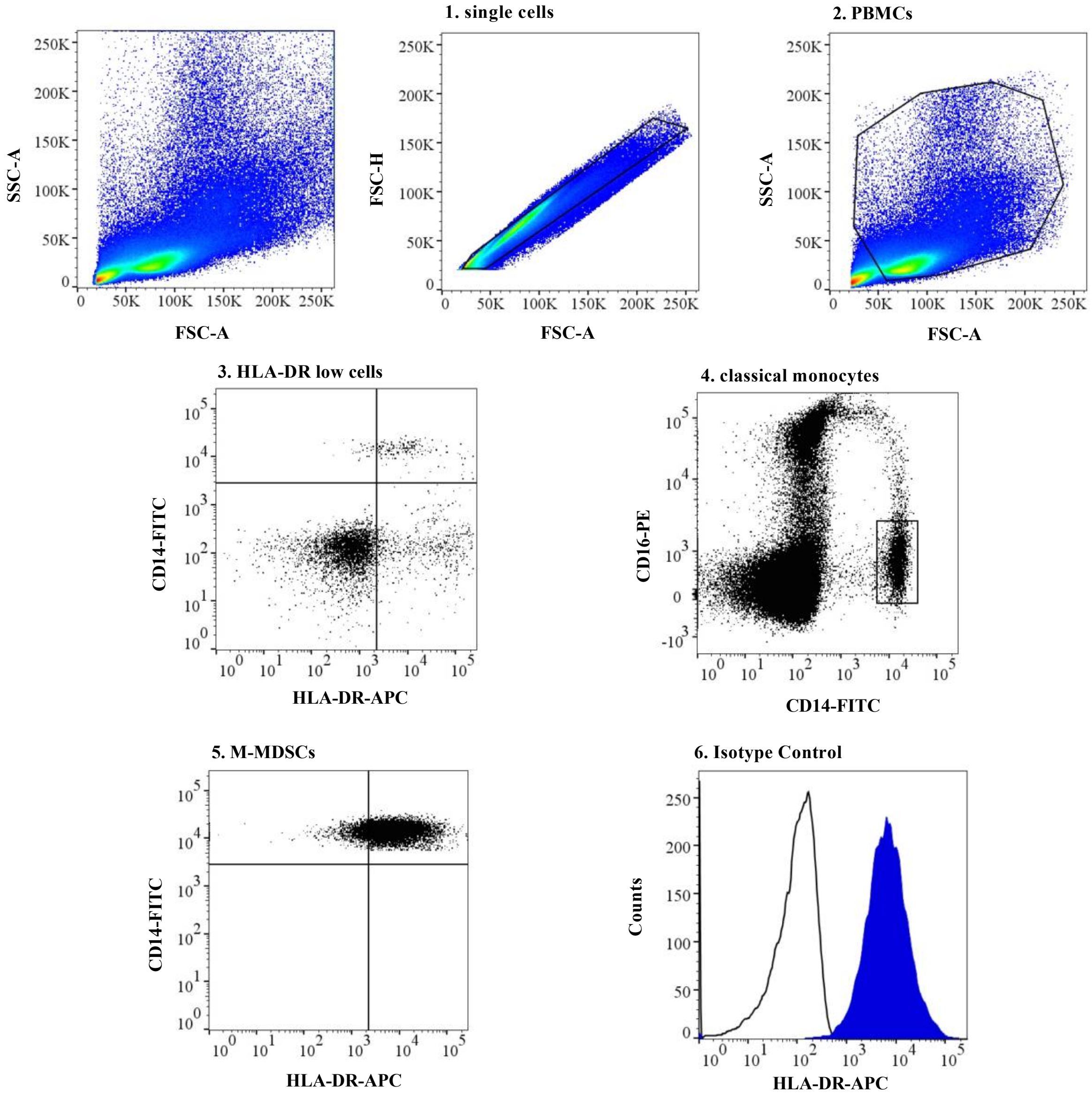

Flow cytometry gating strategy for M-MDSCs: 1.single cells were selected and viable PBMCs were plotted according to their physical parameters, forward and sideward light scatter (2). 3. PBMCs were further subdivided in $CD14^{++}$ and HLA-DR low cells and $CD14^{++}$/ $CD16^{-}$ subsets (4.). M-MDSC were definined as $CD14^{++}$/ $CD16^{-}$ and HLA-DR low cells (5). 6. Histogramm showing $CD14^{++}$/ $CD16^{-}$ cells stained with HLA-DR-APC antibody or isotype control.

Supplementary Figure 3:

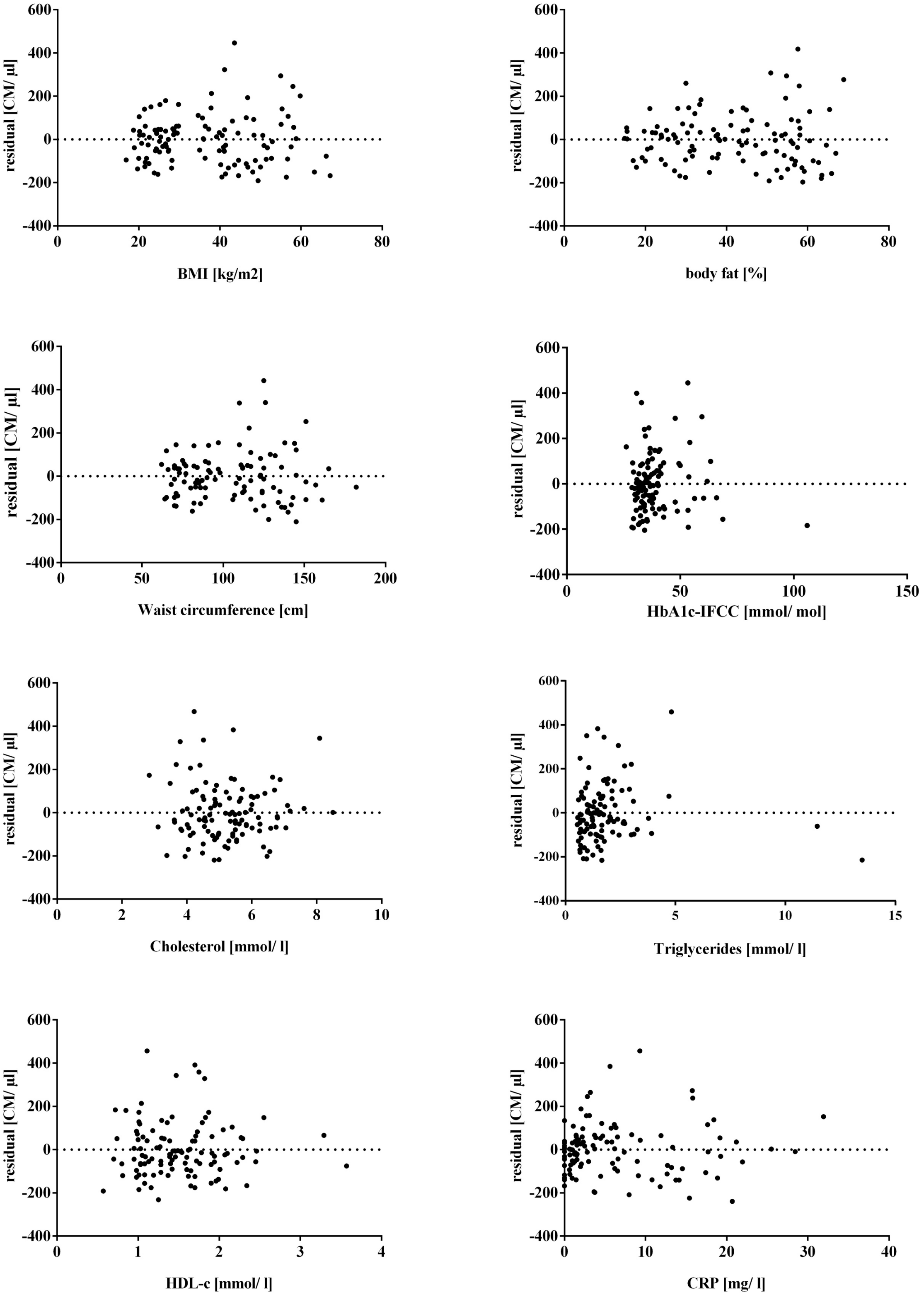

Residual plots of regression analysis of classical monocytes vs. variables of obesity, glucose, lipid metabolism and inflammation in the entire cohort (correlations shown in table 2).

Supplementary Figure 4:

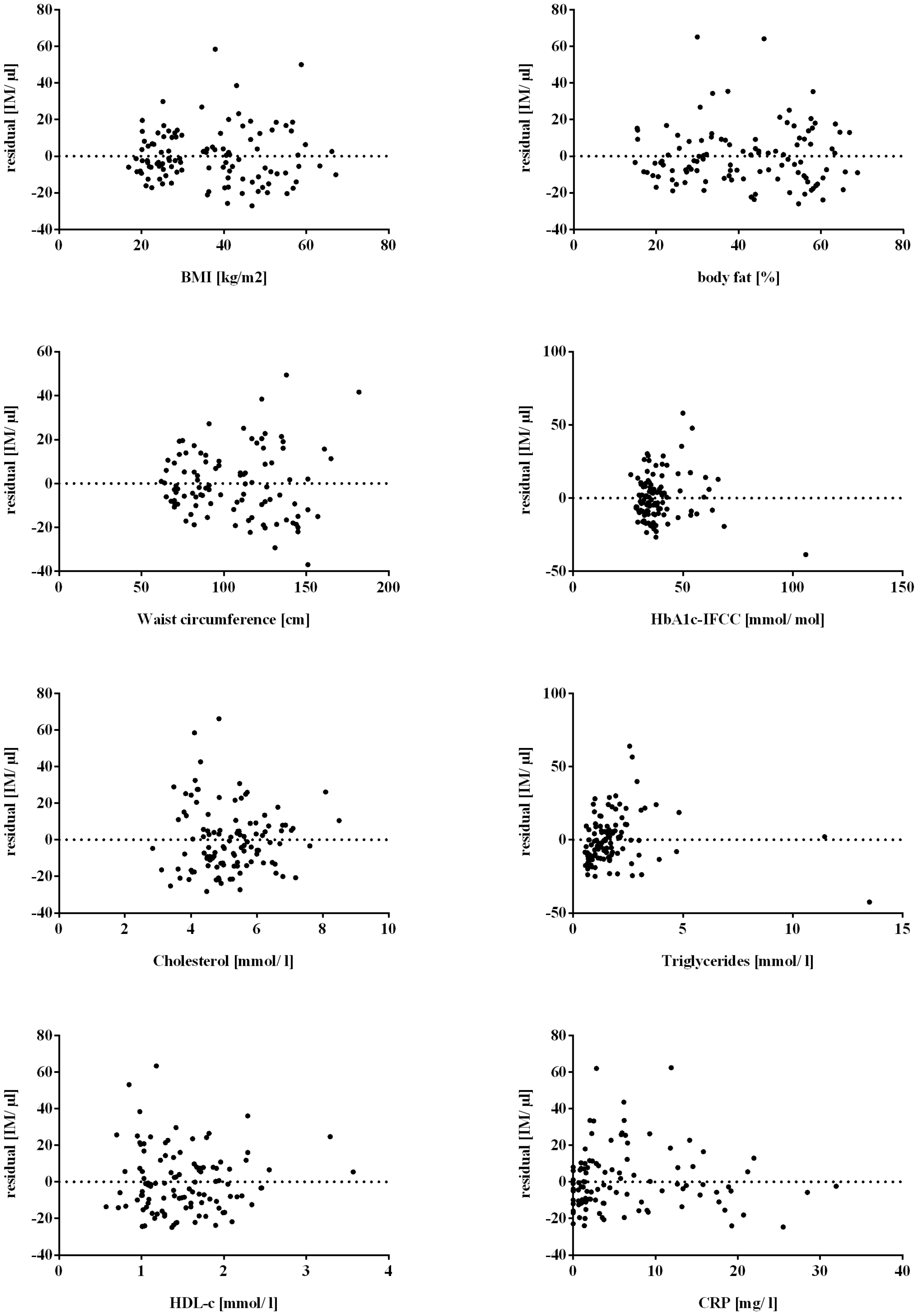

Residual plots of regression analysis of intermediate monocytes vs. variables of obesity, glucose, lipid metabolism and inflammation in the entire cohort (correlations shown in table 2).

Supplementary Figure 5:

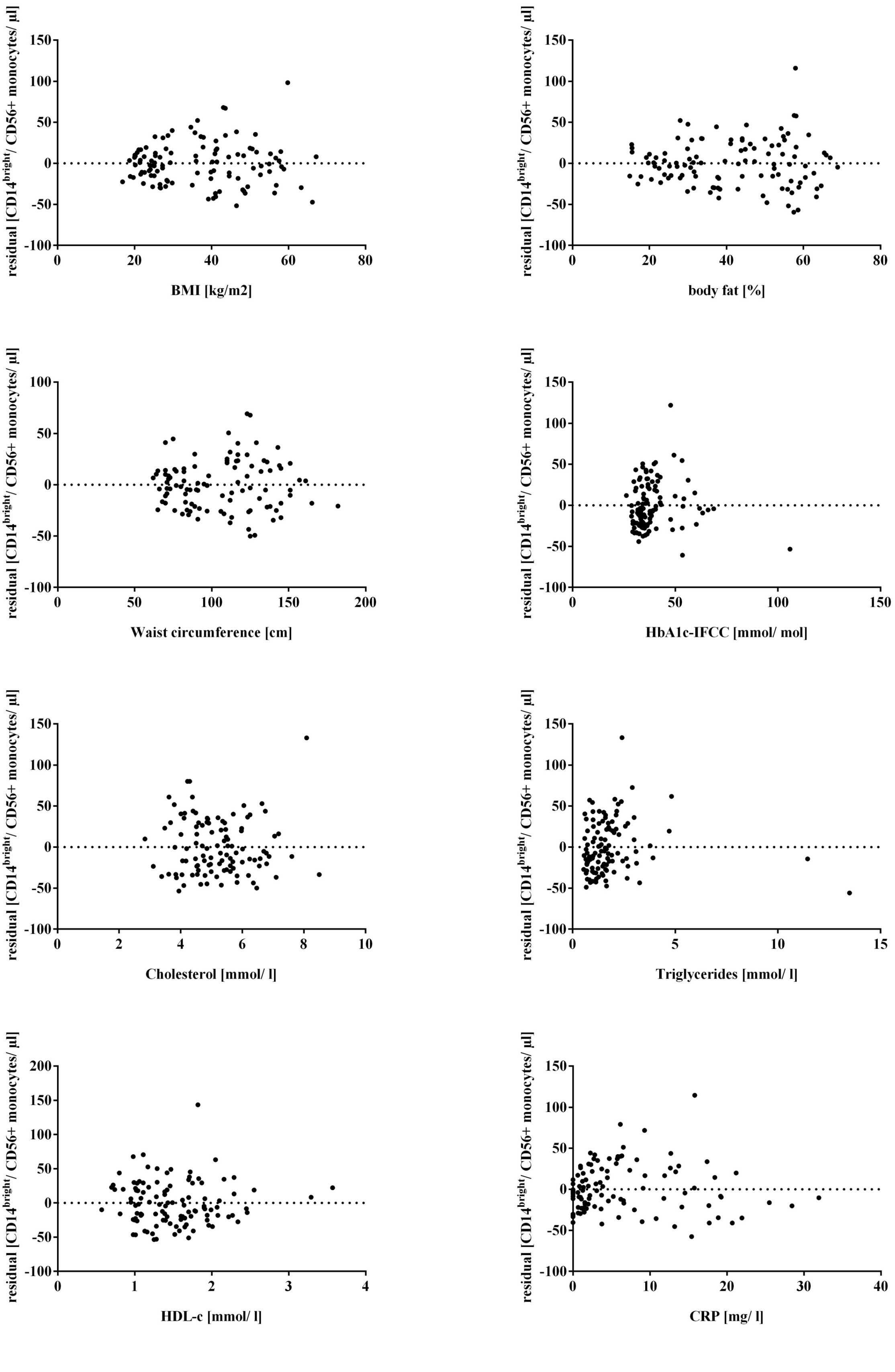

Residual plots of regression analysis of CD56+ monocytes vs. variables of obesity, glucose, lipid metabolism and inflammation in the entire cohort (correlations shown in table 3).

Supplementary Figure 6:

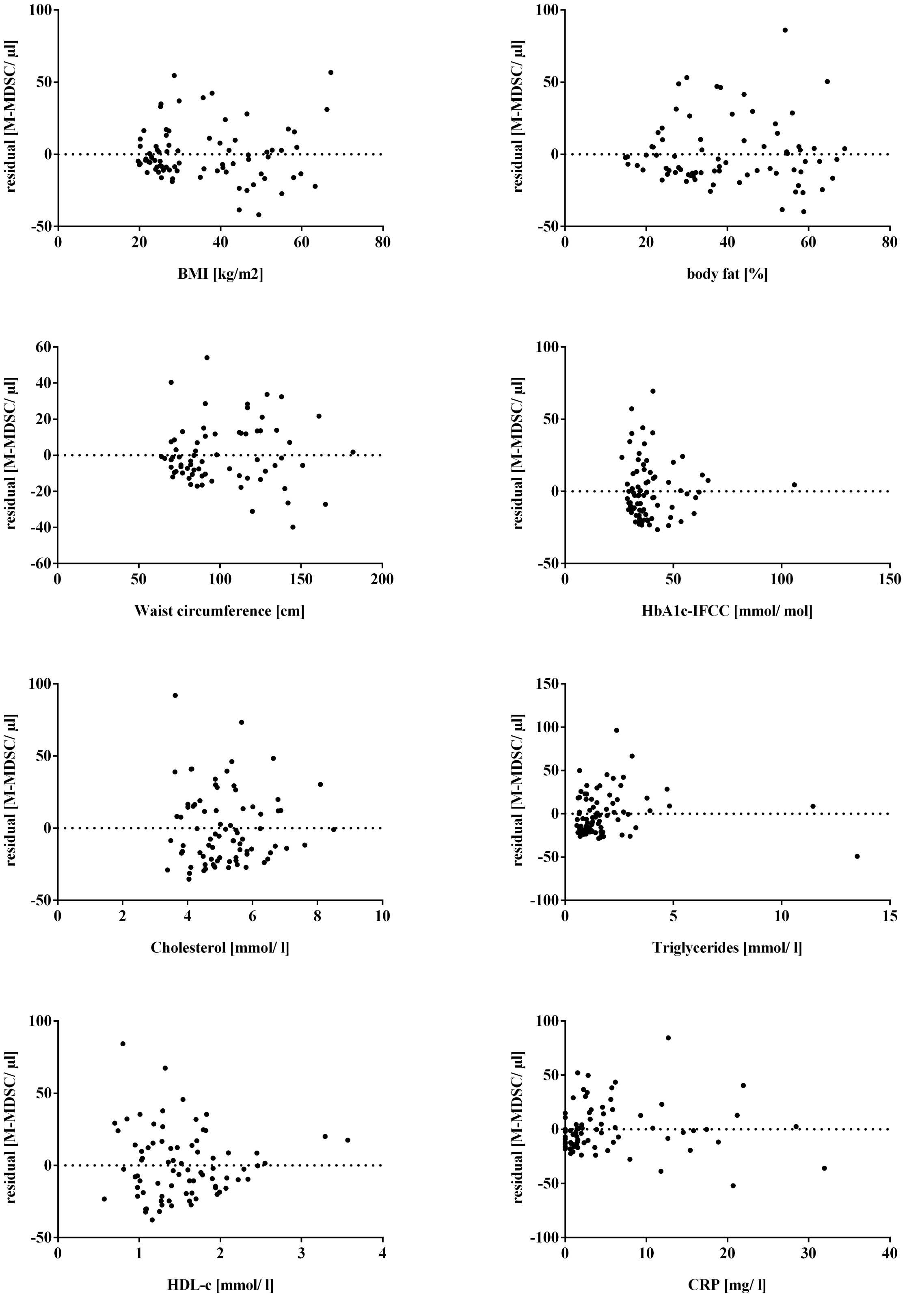

Residual plots of regression analysis of M-MDSC monocytes vs. variables of obesity, glucose, lipid metabolism and inflammation in the entire cohort (correlations shown in table 4).
